# Optimization of Metal Injection Molding Processing Conditions for Reducing Black Lines and Meld Lines in Bone Plates

**DOI:** 10.3390/polym16233241

**Published:** 2024-11-22

**Authors:** Chao-Ming Lin, Po-Yu Yen, Chung-Ming Tan

**Affiliations:** 1 Department of Mechanical and Energy Engineering, National Chiayi University, Chia-Yi 600355, Taiwan; yenboyu@gmail.com; 2 Department of Vehicle Engineering, WuFeng University, Chia-Yi 621303, Taiwan; cmtan@wfu.edu.tw

**Keywords:** bone plate, metal powder injection molding, Taguchi method, grey relational analysis, RMCO, black lines, meld lines

## Abstract

The bone plates used in surgery to assist in fracture healing are often manufactured by metal injection molding (MIM) using a feedstock material consisting of metal powder and polymer binder. However, if the local powder concentration is too low or uneven, black lines may be formed, which impair the product appearance. Furthermore, if the melding temperature is too low, it can lead to meld lines and reduced mechanical properties. Accordingly, this study combines mold flow analysis simulations with the single-objective Taguchi robust design method to determine the MIM processing conditions that optimize the powder concentration and melding temperature. Grey relational analysis (GRA) is then used to establish the processing conditions that simultaneously optimize both MIM objectives. It is found that the processing conditions determined through GRA provide a significant improvement over the original design; however, the experimental outcomes are poorer than those achieved through the single-objective Taguchi experiments since the melt temperature effect suppresses that of all the other processing conditions. Consequently, a robust multi-criteria optimization (RMCO) technique is employed to improve the optimization outcome by identifying the dominant factors in the MIM process and fixing them at optimal levels to redesign the Taguchi experiments to optimize the non-primary factors. It is shown that the RMCO method eliminates interference between the multiple factors and hence provides an improved multi-objective optimization outcome. Overall, the integrated framework proposed in this study advances the optimization of the MIM process for bone plates and leads to improved product quality and performance.

## 1. Introduction

### 1.1. Bone Plates

**Figure 1:**
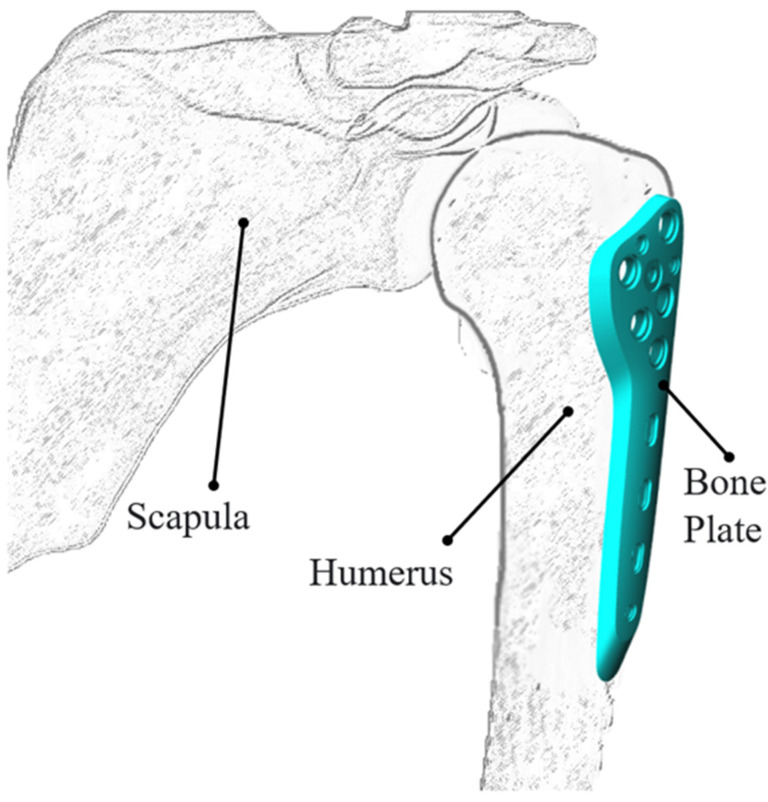
Humeral bone plate.

Bone plates are the most common implants used in internal fixation (Figure 1) and provide multiple advantages, including superior stability, tensile strength, compressive strength, shear strength, torsional strength, and bending resistance for bone fracture implants. Common surgical procedures involving bone plates include open reduction, internal fixation, and bridge plating. Open reduction and internal fixation aim for anatomical reduction but can result in significant damage to the soft tissue and blood supply. Bridge plating techniques cause less soft tissue damage than open reduction but are associated with a higher rate of fracture deformity and an increased risk of localized soft tissue pressure. To address these issues, most modern bone plates incorporate particular design features and locking holes. Surgeons can select the type of screw, such as locking or non-locking, based on the location and morphology of the fracture. However, as medical devices are intended for fixation and support, the precision and strength of the bone plates are critical concerns. Moreover, it is important to balance the need for high-quality and high-performance bone plates with a low fabrication cost and the potential for mass production using standard manufacturing techniques [1,2,3,4,5].

### 1.2. Metal Injection Molding

**Figure 2:**
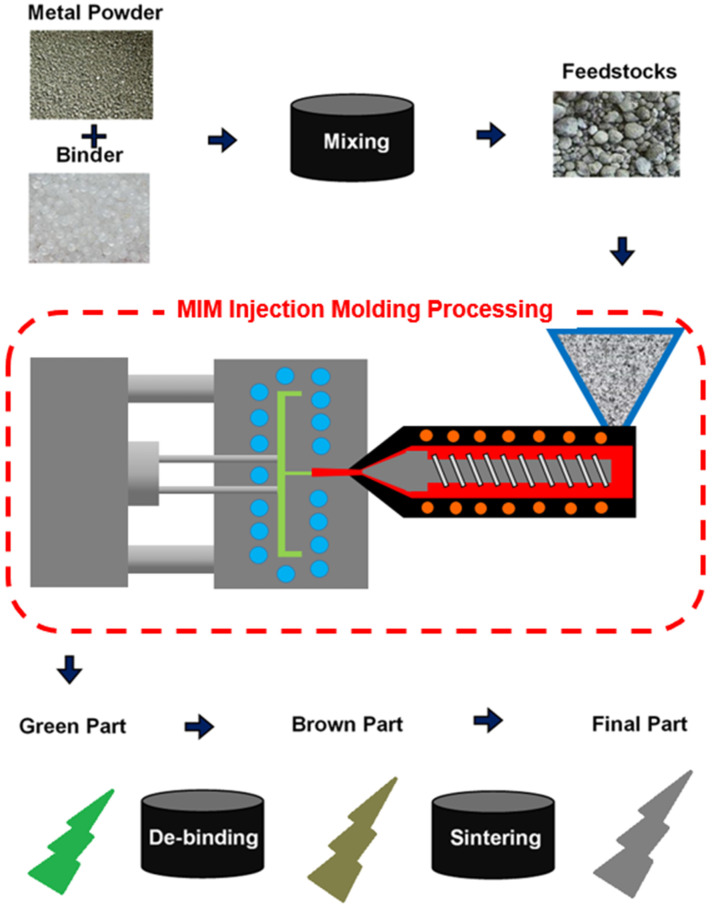
MIM processing flow diagram.

Metal injection molding (MIM), which uses a mixture of fine metal particles and a polymer binder as the feedstock, shares many of the same advantages as plastic molding injection molding, including the ability to produce highly complex geometric designs with large-scale throughput and a consistent quality. The density of the final products can reach as much as 98%, and the tensile strength and sintered density are significantly better than those achieved through traditional powder metallurgy processes. As shown in Figure 2, the feedstock is injected into a mold to produce the desired green part, and the green part then undergoes a de-binding process to become a brown part. Finally, the mechanical properties of the brown part are enhanced through sintering under carefully controlled temperature conditions. The surface smoothness of MIM parts rivals that of metal components finished using conventional mechanical polishing techniques. Furthermore, due to the micron-scale size of the powder particles, the finished parts have high density and superior mechanical properties. Consequently, MIM is now widely used in the manufacture of products and components with high complexity, small size, and refined appearance, such as medical devices, bone plate components, and structural elements of prosthetics [6,7].

**Figure 3:**
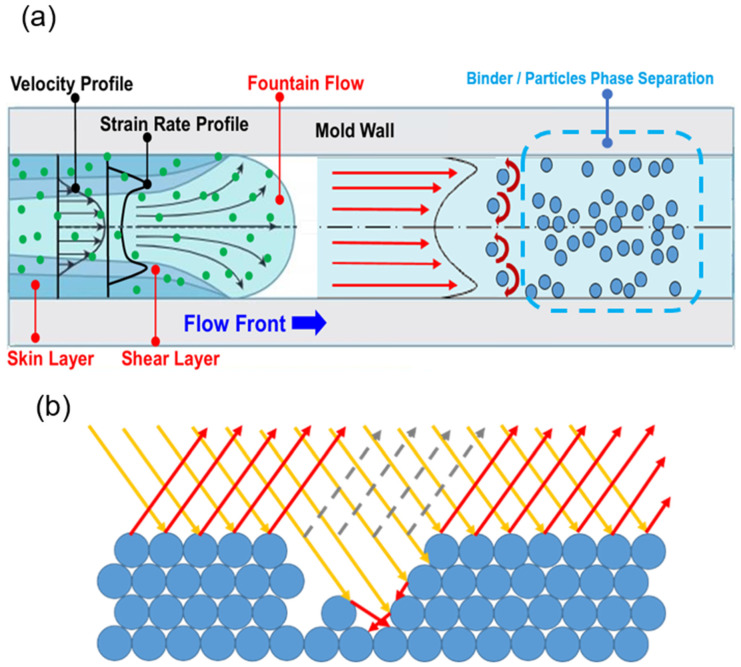
(**a**) Phase separation effects due to shear-rate gradient; (**b**) formation of black lines due to shear-induced phase separation (Notes: The yellow light is the incident light; the red light is the reflected light; the gray dotted line is the dark area showing no reflected light).

However, the MIM process still faces several critical challenges, including compensating for volumetric shrinkage and warpage effects during the cooling phase to ensure that the final component meets the specified requirements for the geometric dimensions and tolerances and improves the aesthetic appearance of the molded part by suppressing the formation of black lines on its surface. Previous studies have indicated [8,9,10] that these lines are caused by the presence of high local shear rates resulting from abrupt changes in the velocity gradient near the surface layer of the mold during the molding process. This high shear rate induces phase separation between the powder particles and the binder, leading to a non-uniform powder particle concentration. The resulting black lines often require subsequent processing for their removal (as shown in Figure 3), thereby increasing the time and cost of the fabrication process.

**Figure 4:**
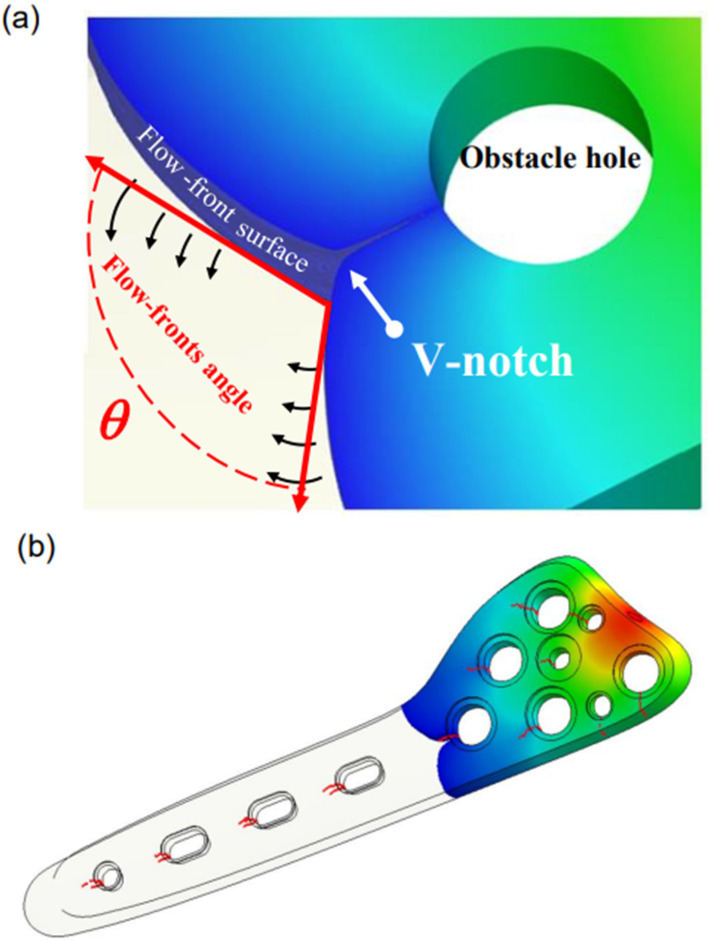
(**a**) V-notch formation as flow-front bypasses obstacle; (**b**) distribution of meld lines (red lines) around bone plate holes. (Note: The color distribution from red to green to blue is the flow-front moving over time.)

As shown in Figure 1, the geometric design of bone plates involves the placement of multiple holes to accommodate screws. However, these holes can create regions of localized stress concentration, which may lead to fracture or deformation of the plate during fabrication or in service. Furthermore, in the molding process, the melt in the mold cavity separates as it flows around these holes, potentially leading to the formation of V-notch effects when the melt flow fonts subsequently recombine (as illustrated in Figure 4a). If the temperature of the two flow fronts is too low, meld lines may form, as shown in Figure 4b. Furthermore, due to the low temperature, the polymer bonds in the binder undergo an incomplete reaction at the junction between the two flows, resulting in a region of weaker strength in the downstream region of the holes. This weakness can cause damage during the de-binding and sintering processes after the green part is formed, which may lead to cracks in the final product and an ultimate reduction in its structural integrity [11,12].

Since MIM products require de-binding and sintering procedures, it is difficult to observe the distribution of the metal powder and defects from the surface of the initial injection parts (i.e., the green or brown parts). In other words, these characteristics can only be properly determined after the sintering process has been completed and the final product has been obtained. Nonetheless, owing to the very small size of most MIM components, direct observation of the metal powder distribution and structural defects in the completed part remains extremely difficult. Thus, to enhance the mechanical and aesthetic quality of MIM products, it is necessary to thoroughly understand the relationship between the key MIM process parameters and the final product performance in advance such that the desired mechanical and aesthetic quality of the MIM product can be achieved through an appropriate setting of the MIM conditions. Experimental trial-and-error methods to determine the optimal MIM processing conditions are time-consuming, expensive, and not guaranteed. Thus, numerical simulation methods combined with various optimization techniques are generally preferred [13,14,15].

### 1.3. Optimization Method to Improve MIM Process

The Taguchi method offers a robust approach to enhance the quality of manufactured products through systematic statistical analysis and a minimal number of experimental trials. Accordingly, in the present study, the Taguchi method is first employed to determine the optimal settings of the key MIM processing conditions (i.e., the gate type, melt temperature, mold temperature, injection speed, packing time, and packing pressure) that maximize the uniformity of the powder concentration distribution and the V-notch melding temperature during the MIM process, thereby suppressing the formation of black lines and meld lines, respectively. However, even though the Taguchi method significantly reduces the number of experiments required to identify the optimal processing conditions, it is a single-objective optimization method. Thus, for the problem considered in the present study, two separate Taguchi experiments must be performed to optimize the processing conditions for the powder concentration distribution and V-notch melding temperature, respectively. Consequently, it is desirable to explore alternative optimization methods capable of producing a comprehensive simulation outcome [16,17,18]. Therefore, the present study utilizes a grey relational analysis (GRA) technique to optimize the MIM processing conditions in such a way as to meet both experimental objectives simultaneously. Finally, a robust multi-criteria optimization (RMCO) method is used to further improve the joint optimization results by identifying the dominant factors in the MIM process and fixing them at optimal levels while further experiments are performed to optimize the levels of the non-primary factors. The experimental results confirm the feasibility of the RMCO method to enhance the product quality and performance of the considered bone plate through an appropriate setting of the MIM processing parameters.

## 2. Theory Analysis, Black Lines and Meld Lines

The mold flow analysis (MFA) simulations conducted in the present study were based on three conservation laws (mass, momentum, and energy) for a generalized Newtonian fluid (GNF), a material constitutive relationship for the MIM feedstock material, and the particle-phase conservation relationship for the rigid spherical particles suspended in the polymer fluid.

### 2.1. Governing Equations

For a GNF, the mass conservation, momentum conservation, and energy conservation equations can be written as [19]

(1) $$\frac{\partial \rho }{\partial t}+\nabla \cdot \left(\rho u\right)=0,$$

(2) $$\frac{\partial (\rho u)}{\partial t}+\nabla \cdot \left(\rho uu-\sigma \right)=\rho g,$$

(3) $$\rho c_{p}\left(\frac{\partial T}{\partial t}+u\cdot \nabla T\right)=\nabla \cdot (k\nabla T)+\eta {\dot{\gamma }}^{2},$$

where u is the velocity, *p* is the pressure, *ρ* is the density, *c_p_* is the heat capacity, *η* is the viscosity, $\dot{\gamma }$ is the shear rate, ***k*** is the heat conductivity, and ***σ*** is the stress tensor (the bulk suspension stress).

### 2.2. Constitutive Equation and Viscosity Mode

For an isotropic fluid, the viscous stress is a function of the local strain rate of the fluid. The rate-of-strain tensor, $\dot{\gamma }$, can be written in a matrix form and the constitutive equation of the polymer fluid then expressed as

(4) $$\sigma =-pI+\tau =-pI+\eta \dot{\gamma }=-pI+\eta (\nabla u+\nabla u^{T})$$

The rheological properties of the polymer melt can be expressed using the modified cross-viscosity model as follows [20]:

(5) $$\eta \left(T,\dot{\gamma }\right)=\frac{\eta_{0}(T)}{1+{\left(\eta_{0}\dot{\gamma }/\tau^{*}\right)}^{1-n}}$$

(6) $$\eta_{0}\left(T\right)=Bexp\left(\frac{T_{b}}{T}+Dp\right),$$

where $\tau^{*}$ is the critical shear stress at the transition from the Newtonian plateau, *n* is the power law index, $\eta_{0}$ is the melt viscosity under zero-shear-rate conditions, *D* is a pressure parameter used to correct for pressure effects on the viscosity, and *p* is the pressure.

### 2.3. Particle-Phase Conservation Equation

The concentration of the metallic powder particles in the polymer binder is described using a suspension balance model, in which the particles are assumed to be rigid bodies and the polymer is a GNF. Moreover, the particle phase is approximated as a pseudo-continuum, and the dominant interactions between the particles involve hydrodynamic, viscous, and non-Brownian forces, with no external field other than gravity. Finally, the Reynolds number is assumed to be negligible [21]. The particle-phase conservation equation is thus written as

(7) $$\frac{\partial \phi }{\partial t}+u\cdot \nabla \phi =-\nabla \cdot \left[\phi \left(u_{p}-u\right)\right],$$

where ϕ is the particle volume fraction, u is the bulk suspension velocity, $\phi \left(u_{p}-u\right)$ is the particle migration flux relative to the bulk motion, and $u_{p}$ is the average particle phase velocity.

By averaging the overall momentum balance across the particle phase and focusing on conditions with low Reynolds numbers as well as neutrally buoyant, monodisperse spheres, the resulting expression for the particle flux is given as follows:

(8) $$\phi \left(u_{p}-u\right)=\frac{2a^{2}}{9\eta_{f}}f\left(\phi \right)\nabla \cdot \sigma_{p},$$

where a is the particle radius, $\eta_{f}$ is the viscosity of the polymer fluid, $f\left(\phi \right)$ is a sedimentation function, and $\sigma_{p}$ is the particle contribution to the bulk stress.

The sedimentation function is written as [22]

(9) $$f\left(\phi \right)=\left(1-\frac{\phi }{\phi_{m}}\right){\left(1-\phi \right)}^{\alpha -1},$$

where $\phi_{m}$ is the maximum packing fraction and α is the index of particle friction.

The particle flux is a cross-stream migration flux of particles that contains both normal and shear stress portions of the particle phase. The particle contribution to the bulk stress, $\sigma_{p},$ can be expressed as [23]

(10) $$\sigma_{p}=2\eta_{f}\cdot \left[{\bar{\eta }}_{s}\left(\phi \right)-1\right]E-\eta_{f}{\cdot \bar{\eta }}_{n}\left(\phi \right)\dot{\gamma }Q,$$

where $\eta_{f}$ is the suspension fluid viscosity, ${\bar{\eta }}_{s}$ is the dimensionless shear viscosity derived with respect to $\eta_{f}$, ${\bar{\eta }}_{n}$ is the dimensionless normal stress viscosity t, E is the rate-of-strain tensor, $\dot{\gamma }=\sqrt{2E:E}$ is the magnitude of the rate-of-strain tensor, and Q is an anisotropic tensor.

The shear and normal stress viscosities are expressed in terms of the dimensionless volume fraction of $\bar{\phi }=\frac{\phi }{\phi_{m}}$, i.e.,

(11) $${\bar{\eta }}_{s}\left(\phi \right)=1+2.5\phi_{m}{\left(1-\bar{\phi }\right)}^{-1}+K_{s}{\bar{\phi }}^{2}{\left(1-\bar{\phi }\right)}^{-2},$$

(12) $${\bar{\eta }}_{n}\left(\phi \right)=K_{n}{\bar{\phi }}^{2}{\left(1-\bar{\phi }\right)}^{-2}$$

where *K_s_* = 0.1 and *K_n_* = 0.75 are factors chosen to match the experimental data of wide-gap Couette flow [24].

The anisotropic tensor is a constant tensor Q, which describes the anisotropy of the normal stresses, i.e.,

(13) $$Q=\left[\begin{array}{ccc} \lambda_{1} & 0 & 0\\ 0 & \lambda_{2} & 0\\ 0 & 0 & \lambda_{3} \end{array}\right],$$

where $\lambda_{1}$, $\lambda_{2}$, and $\lambda_{3}$ are characteristic values for parallel-plate, cone-and-plate, and wide-gap Couette flows.

### 2.4. Shear-Induced Phase Separation Effect

During the MIM process, when a mixed melt containing metallic powder particles randomly distributed within the binder material flows through the gate into the mold, friction between the melt and the cavity walls creates a fountain effect. As shown in Figure 3a, the change in the velocity distribution prompts the formation of a high shear rate region near the channel walls. The resulting steep shear rate gradient leads to a localized rotational effect on the particles. As the melt continues to flow through the channel, the shear rate gradient causes the particles to migrate toward the point of maximum shear, namely, the center of the channel. Consequently, phase separation occurs between the metal powder particles and the binder material, which leads to a reduction in the powder concentration near the mold walls and an increase in the concentration near the center of the channel.

### 2.5. Formation of Black Lines

The variation in powder concentration between the central and peripheral regions of the channel causes the formation of light and dark areas on the surface of the molded product, as illustrated in Figure 3b. In areas where the powder concentration is high, the surface exhibits greater density and smoothness, leading to most of the incident light being reflected, which results in a bright and shiny appearance. In contrast, regions with low powder concentration have reduced surface density and smoothness, which causes partial absorption of the incident light, creating a surface with diminished, uneven gloss and the appearance of dark lines.

### 2.6. Formation of Meld Lines

Meld lines are common defects in injection-molded products. They are not just superficial flaws, as in the case of black lines, but defects that extend throughout the entire area of the joining surfaces. Consequently, they can significantly impact the physical structure of a product. Meld lines can result from the separation and subsequent rejoining of the material flow around cavities or inserts within the mold, from the use of multiple injection gates, or from hesitation or “race-track effects” in areas of the mold with varying wall thickness. If the different flow fronts have cooled before they meet, the polymer bonds at the joining plane of the two fronts may fail to form properly, potentially leading to defects in the molded parts, such as lines, indentations, and/or color variations. Studies have shown that the angle between the velocity vectors at the moment the two flow fronts meet determines the formation of either weld lines (angles less than 135°) or meld lines (angles greater than 135°) [25].

In the case of bone plates containing holes and grooves, the fountain flow effect causes the molecular chains at the front of the two flows that pass around the holes to orient parallel to each other. This further contributes to poor molecular entanglement and prompts the formation of V-shaped micro-notches, as illustrated in Figure 4a. These notches create a structural weakness in the product that causes the molded part to be more prone to fracture or failure during the production and usage phases. Consequently, improving the meld line quality is essential in the design of complex-shaped products. Many methods have been proposed for addressing the V-notch problem, including modifying the mold design, altering the flow path, increasing the product thickness, changing the material, and using fiber reinforcement or low-viscosity materials.

In practice, however, most meld lines are unavoidable due to the gate design and/or mold geometry. As shown in Figure 4b, meld lines are likely to form in all cavities at locations distant from the gate under the effects of local variations in the flow patterns and shear rate distribution. As discussed above, in MIM, the meld lines are formed as a result of an improper bonding of the polymer where the flow fronts meet. This improper bonding, combined with an uneven particle powder concentration near the meld line, can result in a significant impairment of the product strength. Studies have shown that the temperature distribution near the flow fronts during the bonding process can serve as an important indicator of the meld line strength. Thus, the feasibility of suppressing the formation of V-notches by increasing the mold temperature and melt temperature has also attracted significant attention in the literature. [26,27,28,29].

### 2.7. Analysis of Metal Powder Concentration and Temperature Distributions

To prevent the formation of dark lines, it is crucial to minimize the shear rate gradient within the melt flow during the molding process, thereby enhancing the uniformity of the metal powder concentration distribution. In the current MFA simulations, the uniformity of the powder concentration distribution was assessed by determining the standard deviation of the powder particle concentration on the surface mesh, using Equation (14). In particular, the distribution of the metal powder concentration on the surface of the bone plate was divided into multiple intervals, with each interval corresponding to a specific particle volume fraction. Equation (14) was then used to calculate the standard deviation between the local powder concentration and the average powder concentration. A smaller standard deviation indicated a narrower dispersion of the powder concentration and a closer approximation to the mean value (60%). In other words, it signifies a more uniform distribution of the powder particles and hence a lower risk of black line formation.

In evaluating the formation of the meld lines, a similar approach was taken to quantify the average temperature and distribution in the region where the flow fronts of the material met during the molding process.

(14) $$\sigma =\sqrt{\frac{1}{N}\sum_{i=1}^{N}{\left(y_{i}-\mu \right)}^{2}},$$

where *y_i_* is the value of each discrete sampling point, σ is the standard deviation, *μ* is the average value, *N* is the number of sampling points, and *i* is the sampling point index.

## 3. MIM Simulations and Optimization Methods

**Figure 5:**
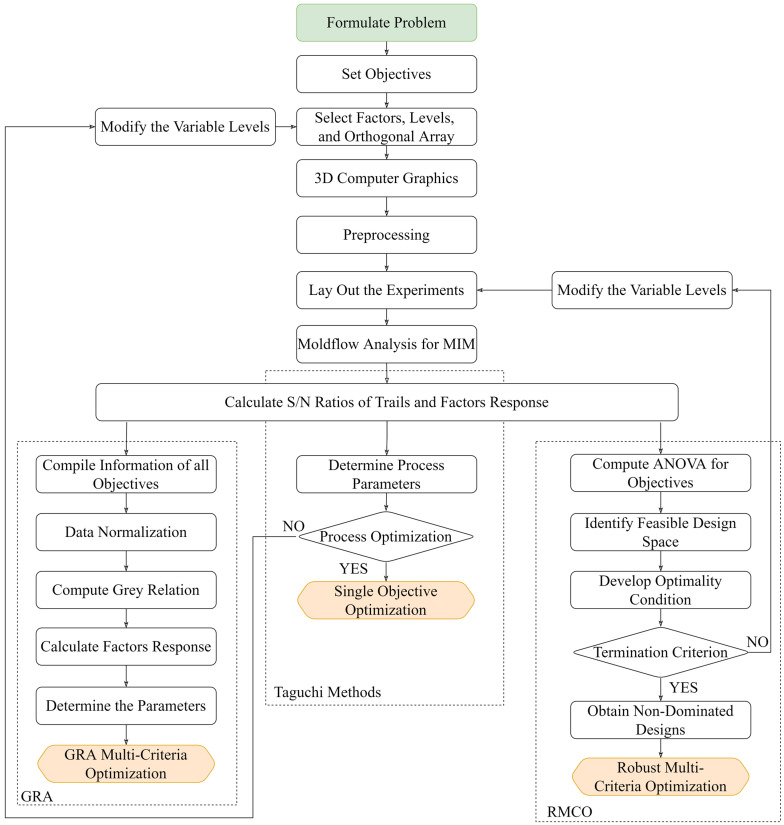
Analytical framework used to optimize MIM processing conditions for bone plate.

The objective of the present study was to determine the MIM processing conditions that minimized the formation of black lines and V-notch meld lines during the bone plate molding process. Figure 5 illustrates the analytical framework. The study commenced by performing Moldex3D mold flow analysis simulations (Core Tech System Co., Ltd., Hsinchu, Taiwan) [30] to evaluate the potential defects that may arise during the manufacturing and usage stages of the bone plate and the temperature distribution near the flow fronts. Based on the MFA results, single-objective Taguchi experiments were conducted to establish the MIM processing conditions that optimized the uniformity of the powder particle distribution and increased the melding temperature. Grey relational analysis (GRA) and robust multi-criteria optimization (RMCO) methods were then applied to perform multi-objective optimization experiments aimed at identifying the processing parameters that simultaneously optimized both the powder particle uniformity and the melding temperature distribution.

### 3.1. Geometry, Material, and Mold Flow Analysis

**Figure 6:**
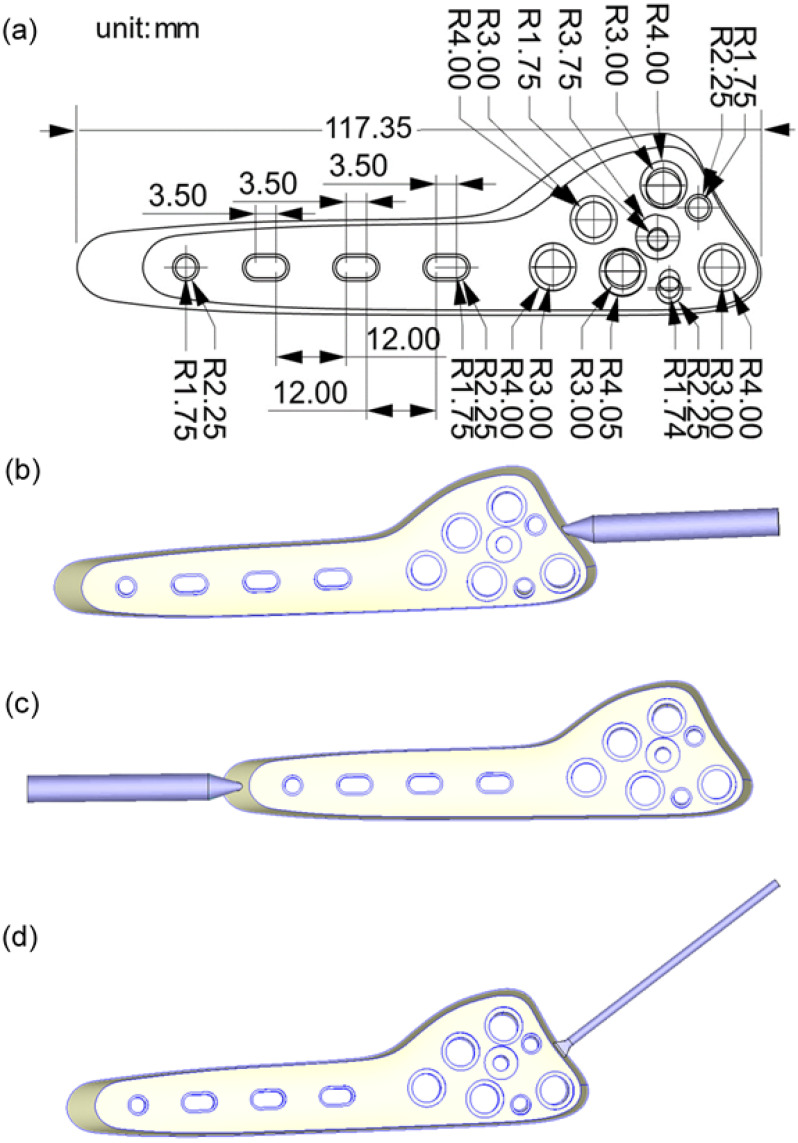
(**a**) Bone plate dimensions (the maximum thickness = 3.931 mm; the average thickness = 3.123 mm); (**b**) injection gate type I; (**c**). injection gate type II; (**d**) injection gate type III.

**Figure 7:**
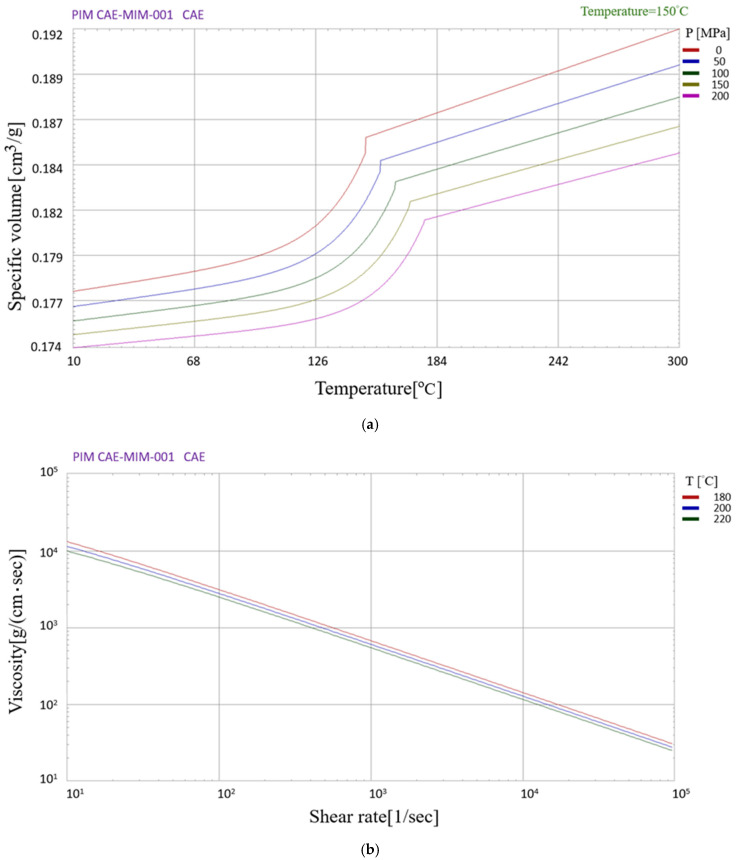
Material properties of MIM feedstock material: (**a**) P-*v*-T diagram; (**b**) viscosity vs. shear rate diagram.

The bone plate model and dimensions are shown in Figure 6a, where the various holes are locking holes used to secure bone screws and other fixation devices [31]. As shown in Figure 6b–d, the analyses considered three different gate systems. The mesh details of the three systems are presented in Table 1. For each meshing scheme, 14,646 measurement points were placed on the surface of the bone plate model to identify the variation in the surface powder concentration caused by the phase separation effect. The bone plate was assumed to be fabricated from CAE-MIM-001 (chosen from the Moldex3D library, Hsinchu, Taiwan), which consisted of a plastic matrix (PP) and metal powder particles (stainless steel) with a volume concentration of 60%. The material information about CAE-MIM-001 used by software developers-Moldex3D is based on their laboratory using a specific manufacturing formula, and the mold flow analysis software (2024) treats the relevant properties obtained from the experiment as a special pure polymer material (mixed formula) for analysis. Because the relative properties (rheological properties, heat transfer properties, and chemical reactions parameters) of the material formula can be measured in the experiment, and the relevant composition of the material itself will not be considered. The mechanical and thermal properties of the composite material and PP binder are listed in Table 2. The thermal-dependent specific volume and viscosity properties are shown in Figure 7a,b, respectively.

### 3.2. Taguchi Experiments

The Taguchi method is a powerful approach for determining optimal processing conditions that ensure the output quality of manufacturing processes is robust against experimental variations. Notably, it maximizes the information gained while minimizing the number of experimental runs, making it particularly useful for optimizing complex processes like MIM, which involve numerous interrelated variables that make traditional trial-and-error methods inefficient, time-consuming, and costly. In the present study, the Taguchi method was employed to identify the optimal MIM processing parameters that enhanced the uniformity of powder particle concentration and resulted in a higher meld line temperature. Typically, when using the Taguchi method, the quality of the outcome from each experimental run is evaluated using a signal-to-noise (S/N) ratio [32]. Different forms of S/N ratios may be applied depending on the problem at hand. In this study, the objective was to minimize the standard deviation metric presented in Equation (14), and therefore the smaller-the-better *S/N* ratio was selected as follows:

(15) $$\frac{S}{N}=-10log\left({\bar{y}}^{2}+{S_{n}}^{2}\right)$$

The simulations considered six control factors: (A) the gate type, (B) the melt temperature, (C) the mold temperature, (D) the injection velocity, (E) the packing time, and (F) the packing pressure. Each factor was assigned five levels, as listed in Table 3. Consequently, the simulations were configured in an L_25_(5^6^) orthogonal array with 25 trials, as shown in Table 4. Based on the recommended processing levels for MIM compound materials specified in the Moldex3D-MIM software, the melt temperature level settings were set in the range of 180 °C to 220 °C; the mold temperature was set between 60 °C and 100 °C; the injection velocity was set between 65 mm/sec and 105 mm/sec; the packing time was set between 4.3 s and 6.3 s; and the packing pressure was set between 50 MPa and 250 MPa.

### 3.3. Grey Relational Analysis (GRA)

Grey relational analysis (GRA) offers a robust solution for addressing multi-objective optimization problems that involve uncertainty, multiple inputs, and incomplete information [33,34,35]. Specifically, GRA models aim to evaluate the correspondence between observed data sequences and reference data to enable reliable predictions about the future development of uncertain systems. Importantly, GRA provides a method for assessing the relative significance of multiple factors in complex, unknown systems and identifying those factors that most significantly impact the quality of the final outcome. The first step in implementing GRA involves preprocessing (or normalizing) the input data to ensure that the performance of all alternatives is compared fairly. In this study, the relationship between the S/N ratios of powder concentration and melding temperature obtained in each run of the Taguchi orthogonal array was quantified using a grey relational grade. This grade was subsequently used as the design criterion in a further Taguchi analysis to determine the optimal control factor and level settings that simultaneously optimized both powder concentration and melding temperature. The original response sequence was normalized as follows:

(16) $${x_{i}}^{*}=\frac{{x_{i}}^{\left(0\right)}\left(k\right)-\min{x_{i}}^{\left(0\right)}(k)}{\max{x_{i}}^{\left(0\right)}(k)-\min{x_{i}}^{\left(0\right)}(k)},$$

where ${x_{i}}^{*}$ is the normalized value from the data, $\min{x_{i}}^{\left(0\right)}(k)$ is the smallest value of ${x_{i}}^{\left(0\right)}\left(k\right)$; and $\max{x_{i}}^{\left(0\right)}(k)$ is the largest value of ${x_{i}}^{\left(0\right)}\left(k\right)$.

In GRA theory, a grey relational coefficient equal to unity implies that the two sequences are completely related. The equations are given as follows:

(17) $${∆}_{ij}\left(k\right)=\left\|x_{i}\left(k\right)-x_{j}\left(k\right)\right\|,$$

(18) $$\gamma \left(x_{i}\left(k\right),x_{j}\left(k\right)\right)=\frac{∆min+\delta ∆max}{{∆}_{ij}\left(k\right)+\delta ∆max},$$

where *i* = 1, 2, 3, …, *m*; *j* = 1, 2, 3…, *m*; *k =* 1, 2, 3, …, *n*; ${∆}_{ij}\left(k\right)$ is the absolute value difference between $x_{i}\left(k\right)$ and $x_{j}\left(k\right)$; $x_{i}\left(k\right)$ is the reference series; $x_{j}\left(k\right)$ is a specific comparison series; $\gamma \left(x_{i}\left(k\right),\;{\;x}_{j}\left(k\right)\right)$ is the grey relational coefficient; Δ_min_ is the smallest value; Δ_max_ is the largest value; and δ is the distinguishing coefficient, where $\delta \in \left[0,\;1\right]$. (Note that the distinguishing coefficient is an index used to distinguish among the factors and was assigned a value of 0.5 by default in the present study.)

Having calculated all the grey relational coefficients, the corresponding grey relational grades were obtained as follows:

(19) $$\Gamma \left(x_{i},x_{j}\right)=\frac{1}{c}\sum_{k=1}^{c}\gamma \left(x_{i}\left(k\right),x_{j}\left(k\right)\right)$$

where $\Gamma \left(x_{i},\;x_{j}\right)$ is the grey relational grade and *c* is the number of sequences.

### 3.4. Robust Multi-Criteria Optimization (RMCO)

The robust multi-criteria optimization (RMCO) method utilizes a Pareto-optimal method to modify an original experimental design. Essentially, RMCO uses the analysis of variance (ANOVA) results obtained from the original experimental design to quantify the contributions of the individual design factors and then obtains the best solutions for the main factors and non-main factors, respectively. In the present study, RMCO is employed to refine the experimental groups established in the Taguchi analysis, ultimately selecting the appropriate factor levels or reducing the design dimensions based on the following optimization rules: (1) If a factor has a significant effect on all the objective functions, optimization is performed on all levels that contain at least one of the objectives. (2) If a factor has a significant effect on a single objective, the level of the factor that optimizes that objective is chosen, regardless of its importance to the other objectives. (3) If a factor does not significantly affect any of the objectives, the objective most affected by that factor is determined, and the optimal level for that factor is selected by the experimenter [36,37,38,39,40].

## 4. Results and Discussion

### 4.1. Single-Objective Optimization with Taguchi Method

The aim of the Taguchi experiments was to determine the control factor level settings that yielded the smallest standard deviation (Equation (14)) by most closely approaching the average powder particle concentration and melding temperature, thereby minimizing the occurrence of black lines and meld lines. The rightmost columns in Table 4 show the S/N ratios obtained for the powder particle concentration (S/N_1_) and melding temperature (S/N_2_) in each run of the L_25_(5^6^) orthogonal array (OA).

Table 5 shows the Taguchi response results when taking the powder concentration quality as the sole optimization objective. It can be seen that the gate type is the primary influencing factor (66.7%), and the melt temperature is the secondary factor (26.1%). The remaining factors have only minor impacts on the powder concentration distribution (<2.7%). These findings are reasonable since the gate type dominates the flow rate of the polymer melt entering the mold cavity and hence determines the shear effects of the flow and the resulting phase separation. Additionally, the melt temperature affects the fluid viscosity, which also indirectly influences the flow rate. In particular, the solidified layer near the mold surface, caused by friction, creates a velocity gradient, which leads in turn to a shear rate gradient that promotes phase separation. This phase separation leads to a lower concentration of powder particles at the surface and results in unevenness, ultimately causing the formation of black lines. However, as the melt temperature increases, the velocity gradient near the mold surface reduces. As a result, the phase separation effect also reduces, and hence the powder concentration uniformity improves.

Table 6 shows the Taguchi response results obtained when taking the meld line formation as the sole optimization objective. The results indicate that meld line formation is determined mainly by the melding temperature at the point of contact between the two flow fronts (71.7%). All the other factors have only minor effects (<8.9%). Again, this finding is reasonable since, when the temperature is higher, the polymer binder remains in a more fluid state, allowing better molecular entanglement and an improved fusion of the flow fronts.

**Figure 8:**
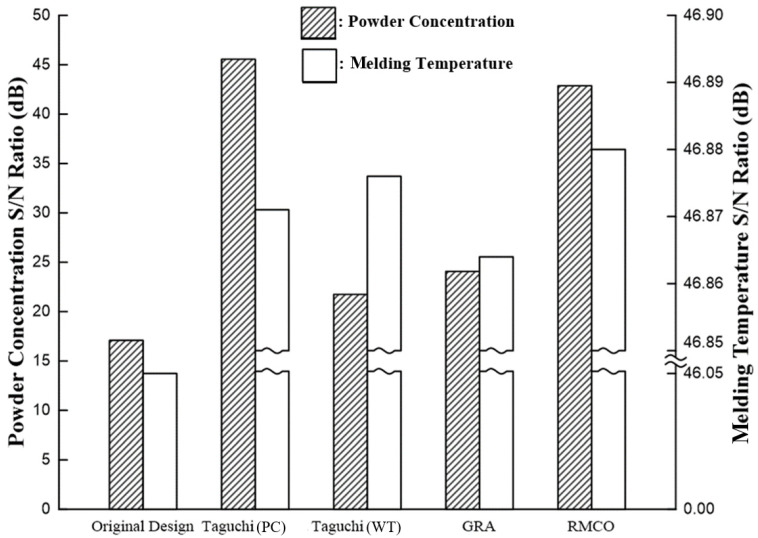
S/N ratios obtained for powder concentration and melding temperature by different optimization methods.

As shown in Figure 8, the single-objective optimization experiments successfully improve the powder concentration and melding temperature compared with the original design. In addition, the response results in Table 5 and Table 6 show that the melt temperature has a significant effect on both quality factors, in particular the melding temperature. By contrast, the gate type has a significant impact on the powder concentration but has no significant effect on the melding temperature. Thus, to optimize both objectives simultaneously, grey relational analysis (GRA) was performed, as discussed below.

### 4.2. Two-Objective Optimization with GRA

The S/N ratios in the two rightmost columns of Table 4 for the powder concentration and melding temperature, respectively, were processed using GRA to identify the set of processing parameters that optimized both objectives simultaneously. Table 7 presents the GRA results, where the quality outputs are normalized into grey relational coefficients with values ranging from 0 to 1. The GRA response table shown in Table 8 indicates that the melt temperature has a significant influence (73.3%) on the possibility of simultaneously optimizing both objectives, followed by the gate type (26.4%). By contrast, the remaining factors have only a minimal impact (<5.5%).

The results presented in Figure 8 show that even though the powder concentration uniformity and melding temperature obtained using the GRA method are better than those obtained using the original design, they are inferior to those obtained in the single-objective Taguchi experiments. This outcome may be attributed to the large disparity in the influence of the different control factors on the individual quality objectives (as discussed in Section 4.1). Given the less-than-satisfactory results obtained in the GRA analysis, the RMCO method was applied in an attempt to further improve the multiple-objective optimization outcome.

### 4.3. Optimization Analysis Results from RMCO

The single-objective Taguchi optimization results (Table 5 and Table 6) showed that the melt temperature was the dominant factor in optimizing the meld-line temperature and a secondary factor in optimizing the powder concentration. According to Rule 2 of the RMCO method and the Pareto optimality principle, the melt temperature was fixed at Level 5, and the Taguchi experiments were redesigned (Table 9) to optimize the non-primary factors. The results (Table 10) showed that the optimal solution for the powder concentration was obtained using the Group 11 parameter settings. Similarly, for the melding temperature, the optimal parameters were those specified for Group 9. However, because the difference in S/N values optimized at melding temperature is small, Group 11 is designated as the RMCO optimized processing parameter combination. The results presented in Table 11 and Figure 8 confirm that the RMCO method significantly improves both the powder concentration and the melding temperature compared to the original design. Furthermore, the melding temperature is higher than that obtained in both the Taguchi experiments and the GRA experiments, while the powder concentration is also higher than that in the GRA experiments and very close to that in the Taguchi experiments.

### 4.4. Improvement Performance Analysis

**Figure 9:**
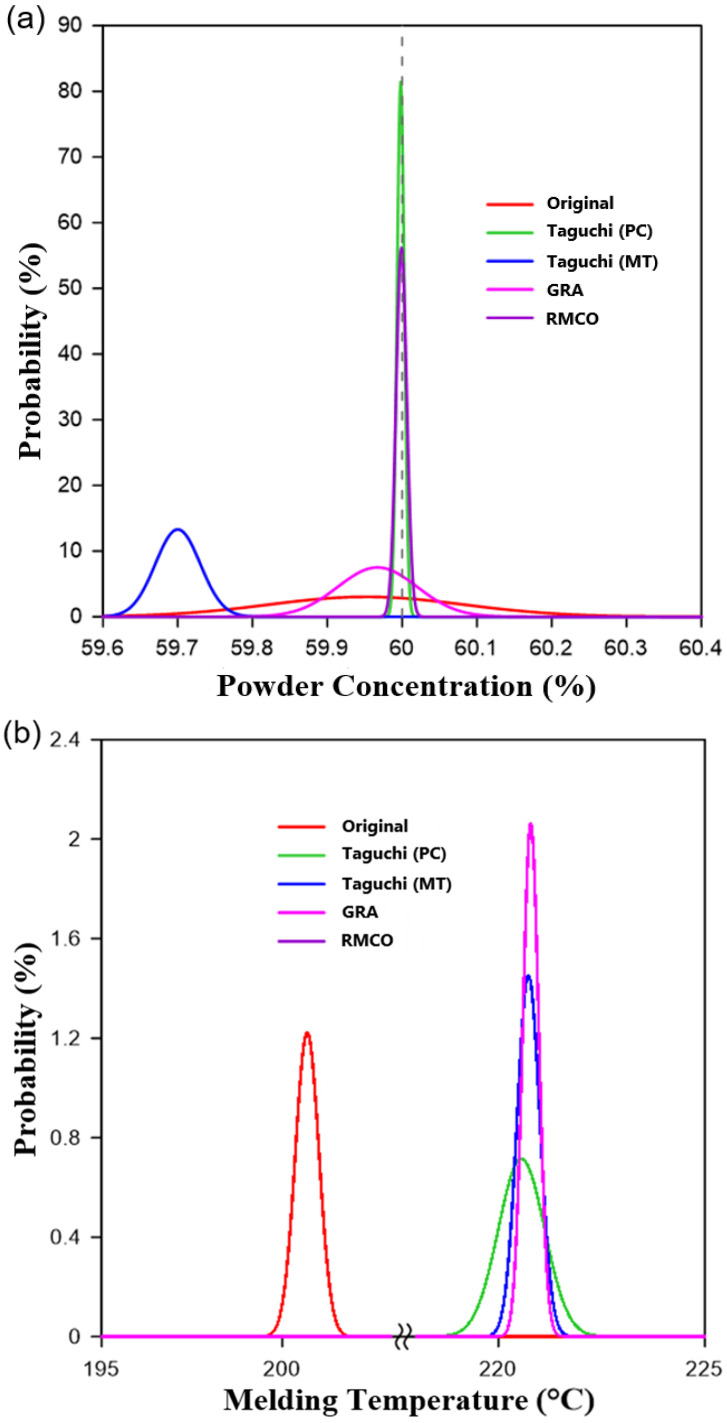
Probability distribution diagrams of different optimization methods: (**a**) powder concentration; (**b**) melding temperature. (Note that Taguchi PC curve and RMCO curves are nearly coincident).

**Figure 10:**
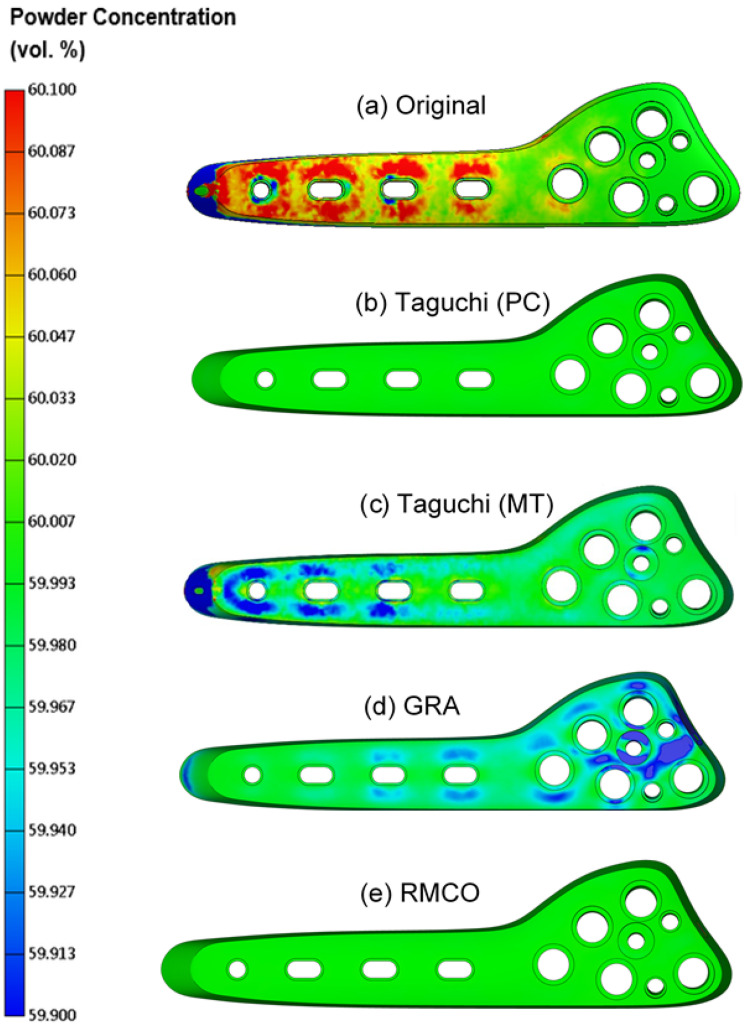
Powder concentration improvement in different methods: (**a**) original; (**b**) Taguchi powder concentration; (**c**) Taguchi melding temperature; (**d**) GRA; (**e**) RMCO.

Figure 9a shows the distribution of the powder particle concentration at the bone plate surface for each of the four considered optimization methods (Use normal distribution for comparison). The mean and standard deviation values of each distribution are listed in Table 11. The original design exhibits a flat distribution, indicating a poor uniformity, and has a peak value lower than 60%, which indicates a poor average concentration. The average powder particle concentration and uniformity are both significantly improved by the Taguchi method aimed at optimizing the powder concentration, grey relational analysis, and RMCO, respectively. However, as expected, the Taguchi method aimed at optimizing the melding temperature leads to a poor average powder particle concentration and uniformity. The superior performance of the RMCO and Taguchi (powder concentration) methods in suppressing the formation of black lines on the bone plate surface is shown in Figure 10a–e.

**Figure 11:**
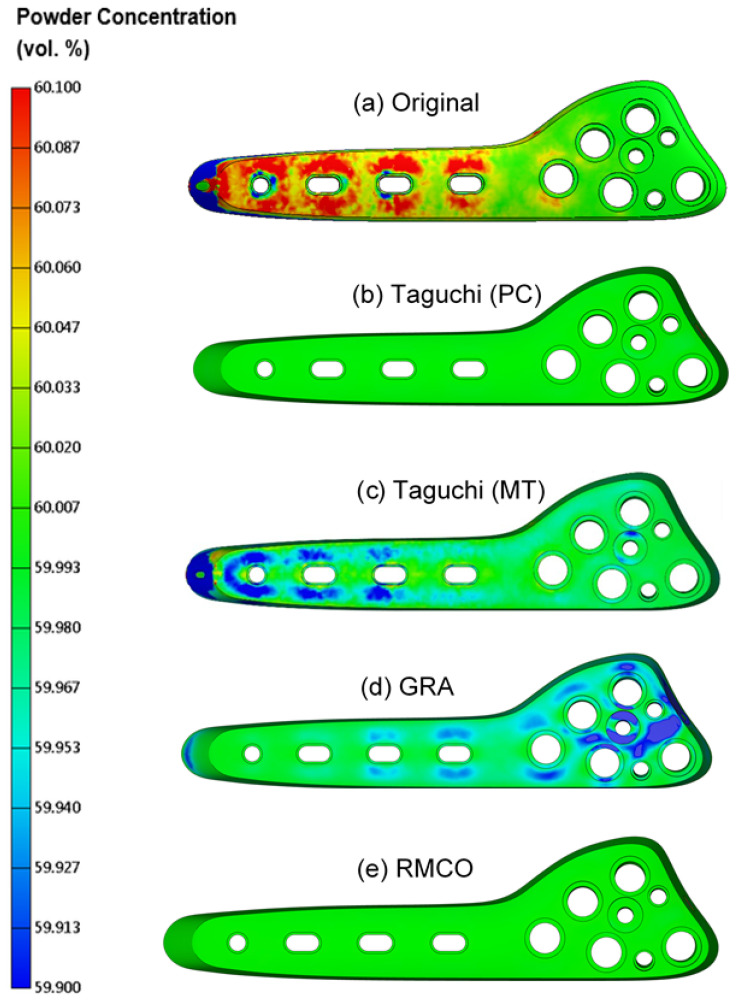
Melding temperature improvement in different methods: (**a**) original; (**b**) Taguchi powder concentration; (**c**) Taguchi melding temperature; (**d**) GRA; (**e**) RMCO.

Figure 9b shows the melding temperature distributions obtained under each of the four optimization methods (Use normal distribution for comparison). The mean and standard deviation values of each distribution are listed in Table 11. The original design results in a relatively low meld line temperature of 200.69 °C. By contrast, all four optimization methods achieve a higher melding temperature of approximately 220 °C. The success of the four methods in improving the meld-line distribution on the bone plate surface, thereby reducing the V-notching effect, is shown in Figure 11a–e.

## 5. Conclusions

This study integrates MFA simulations with three optimization methods—Taguchi experimental design, grey relational analysis (GRA), and RMCO—to identify the processing parameters that minimize the formation of black lines and meld lines in the MIM fabrication of bone plates. The findings demonstrate that these optimization methods significantly outperform the original design in enhancing powder particle concentration on the bone plate surface (thereby reducing black line formation) and increasing meld line temperature (thereby suppressing V-notch defects). Among the methods, the Taguchi approach excels in single-objective optimization, while GRA achieves notable dual-objective improvements but is limited by the dominant influence of melt temperature, which suppresses the effects of other parameters. RMCO outperforms both methods in melding temperature and achieves powder concentration levels comparable to the Taguchi method. These results have significant practical implications, as optimizing powder concentration and melding temperature can substantially reduce defects, producing stronger and more reliable implants. Future research may explore multi-objective optimization by considering additional quality factors—such as deformation, warpage, shrinkage, and strength—while incorporating more processing factors and levels in numerical simulations of mold flow analysis.

## Figures and Tables

**Table 1:** Numerical mesh analysis details.

	Type I	Type II	Type III
Part element	377,600	376,278	372,889
Node number	401,795	407,103	448,419
Measure node	14,646	14,646	14,646

**Table 2:** MIM feedstock material composition and properties.

Property	PP with 60% vol. Stainless Steel Powder	PP
*ρ*: density [g/cm^3^]	5.1	0.91
*μ*: viscosity [Pa-s]	(3.5~5.5) × 10^2^ (T: 190–230 °C; $\dot{\gamma }:\;$10–10^5^)	(6.8~9) × 10^2^ (T: 190–270 °C; $\dot{\gamma }:\;$10–10^5^)
*k*: heat conduction [erg/(s·cm·K)]	8.5 × 10^−5^	1.5 × 10^−4^
*α*: thermal expansion [1/K]	8 × 10^4^	2.52 × 10^4^
*C_p_*: specific heat [erg/(g·K)]	8 × 10^6^	3.1 × 10^7^
*C_v_*: specific volume [cm^3^/g]	0.183–0.201 (T: 10–300 °C; P: 0–200 MPa)	0.777–0.974 (T: 10–300 °C; P: 0–200 MPa)

**Table 3:** Control factors and level settings for single-objective Taguchi experiments.

	A	B	C	D	E	F
	Gate Type	Melt Temp.	Mold Temp.	Injection Velocity	Packing Time	Packing Pressure
		(℃)	(℃)	(mm/s)	(s)	(250 MPa)
Level 1	Type I	180	60	65	4.3	20%
Level 2	Type II	190	70	75	4.8	40%
Level 3	Type III	200	80	85	5.3	60%
Level 4	Type I	210	90	95	5.8	80%
Level 5	Type II	220	100	105	6.3	100%

**Table 4:** L_25_(5^6^) orthogonal array for Taguchi single-objective optimization experiments for powder concentration and melding temperature.

Trials	A	B (°C)	C (°C)	D (mm/s)	E (s)	F (%)	S/N_1_ Powder Concentration	S/N_2_ Melding Temperature
1	Type I	180	60	65	4.3	20	12.82	42.78
2	Type I	190	70	75	4.8	40	18.98	45.60
3	Type I	200	80	85	5.3	60	28.90	46.04
4	Type I	210	90	95	5.8	80	27.88	46.46
5	Type I	220	100	105	6.3	100	32.49	46.74
6	Type II	180	70	85	5.8	100	12.09	45.16
7	Type II	190	80	95	6.3	20	15.70	45.62
8	Type II	200	90	105	4.3	40	19.33	46.07
9	Type II	210	100	65	4.8	60	18.46	46.46
10	Type II	220	60	75	5.3	80	21.74	46.87
11	Type III	180	80	105	4.8	80	32.96	45.15
12	Type III	190	90	65	5.3	100	27.64	45.56
13	Type III	200	100	75	5.3	20	39.47	46.05
14	Type III	210	60	85	6.3	40	47.61	46.47
15	Type III	220	70	95	4.3	60	42.94	46.87
16	Type I	180	90	75	6.3	60	14.28	45.13
17	Type I	190	100	85	4.3	80	17.96	45.68
18	Type I	200	60	95	4.8	100	28.69	46.05
19	Type I	210	70	105	5.3	20	28.21	46.55
20	Type I	220	80	65	5.8	40	34.18	46.51
21	Type II	180	100	95	5.3	40	13.14	45.80
22	Type II	190	60	105	5.8	60	16.48	45.63
23	Type II	200	70	65	6.3	80	15.03	46.05
24	Type II	210	80	75	4.3	100	21.80	46.87
25	Type II	220	90	85	4.8	20	22.30	46.87
Origin	-	-	-	-	-	-		46.05

Hints: Original design conditions. 1. A = Type II, B = 200 °C, C = 80 °C; 2. Three grades injection velocity: 48.9 mm/s, 106.6 mm/s, and 79.9 mm/s; 3. Screw positions: 24.9 mm, 21.0 mm, and 2.1 mm; 4. Packing time: 5.3 s; packing pressure: 70%.

**Table 5:** Taguchi responses for powder concentration.

	Level 1	Level 2	Level 3	Level 4	Level 5	Range	Rank	SS	DOF	%Cont.
A	24.440	17.607	38.122				1	1818.63	4	66.7%
B	17.057	19.350	26.286	28.794	30.729	13.672	2	711.66	4	26.1%
C	25.468	23.450	26.708	22.286	24.304	4.4219	3	59.199	4	2.2%
D	21.627	23.251	25.772	25.671	25.895	4.2684	4	73.674	4	2.7%
E	22.969	24.277	23.926	26.019	25.023	3.0502	6	26.445	4	1.0%
F	23.702	26.648	24.211	23.112	24.542	3.5359	5	36.234	4	1.3%

**Table 6:** Taguchi responses for melding temperature.

	Level 1	Level 2	Level 3	Level 4	Level 5	Range	Rank	SS	DOF	%Cont.
A	45.755	46.139	46.020			0.384	6	0.644	4	3.7%
B	44.804	45.618	46.050	46.564	46.772	1.968	1	12.430	4	71.7%
C	45.558	46.045	46.038	46.019	46.148	0.590	3	1.068	4	6.2%
D	45.472	46.104	46.045	46.160	46.027	0.688	2	1.551	4	8.9%
E	45.653	46.026	46.165	45.961	46.003	0.512	5	0.712	4	4.1%
F	45.577	46.090	46.026	46.041	46.074	0.513	4	0.939	4	5.4%

**Table 7:** Grey relational analysis for powder concentration and melding temperature.

Trials	Powder Concentration				MeldingTemperature			
	S/N	Xi(k)	△i(k)	γ(k)	S/N	Xi(k)	△i(k)	γ(k)
1	12.82	0.02	0.98	0.33798	42.78	0.00	1.00	0.33333
2	18.98	0.19	0.81	0.38281	45.60	0.69	0.31	0.61603
3	28.90	0.47	0.53	0.48700	46.04	0.80	0.20	0.71088
4	27.88	0.44	0.56	0.47375	46.46	0.90	0.10	0.83314
5	32.49	0.57	0.43	0.54018	46.74	0.97	0.03	0.93944
6	12.09	0.00	1.00	0.33333	45.16	0.58	0.42	0.54353
7	15.70	0.10	0.90	0.35758	45.62	0.69	0.31	0.62045
8	19.33	0.20	0.80	0.38578	46.07	0.80	0.20	0.71695
9	18.46	0.18	0.82	0.37861	46.46	0.90	0.10	0.83290
10	21.74	0.27	0.73	0.40702	46.87	1.00	0.00	0.99657
11	32.96	0.59	0.41	0.54789	45.15	0.58	0.42	0.54237
12	27.64	0.44	0.56	0.47067	45.56	0.68	0.32	0.60905
13	39.47	0.77	0.23	0.68561	46.05	0.80	0.20	0.71345
14	47.61	1.00	0.00	1.00000	46.47	0.90	0.10	0.83564
15	42.94	0.87	0.13	0.79158	46.87	1.00	0.00	0.99728
16	14.28	0.06	0.94	0.34761	45.13	0.57	0.43	0.54033
17	17.96	0.17	0.83	0.37457	45.68	0.71	0.29	0.63171
18	28.69	0.47	0.53	0.48421	46.05	0.80	0.20	0.71178
19	28.21	0.45	0.55	0.47798	46.55	0.92	0.08	0.86478
20	34.18	0.62	0.38	0.56938	46.51	0.91	0.09	0.84898
21	13.14	0.03	0.97	0.34004	45.80	0.74	0.26	0.65642
22	16.48	0.12	0.88	0.36326	45.63	0.69	0.31	0.62112
23	15.03	0.08	0.92	0.35282	46.05	0.80	0.20	0.71219
24	21.80	0.27	0.73	0.40762	46.87	1.00	0.00	0.99766
25	22.30	0.29	0.71	0.41236	46.87	1.00	0.00	1.00000

**Table 8:** GRA responses.

	Level 1	Level 2	Level 3	Level 4	Level 5	Range	Rank	SS	DOF	%Cont.
A	0.575	0.572	0.719			0.148	1	0.118	4	26.4%
B	0.452	0.505	0.596	0.710	0.750	0.144	2	0.328	4	73.3%
C	0.609	0.607	0.609	0.579	0.609	0.002	6	0.004	4	0.8%
D	0.545	0.609	0.633	0.627	0.600	0.088	3	0.025	4	5.5%
E	0.597	0.591	0.602	0.599	0.625	0.011	5	0.003	4	0.7%
F	0.580	0.635	0.607	0.587	0.604	0.055	4	0.009	4	2.0%

**Table 9:** RMCO-modified control factors and level settings.

	A	B	C	D	E	F
	Gate Type	Melt Temp.	Mold Temp.	Injection Velocity	Packing Time	Packing Pressure
		(℃)	(℃)	(mm/sec)	(s)	(250 MPa)
Level 1	Type I	220	60	65	4.3	20%
Level 2	Type II	220	70	75	4.8	40%
Level 3	Type III	220	80	85	5.3	60%
Level 4	Type I	220	90	95	5.8	80%
Level 5	Type II	220	100	105	6.3	100%

**Table 10:** RMCO analysis results.

Trials	A (-)	B (°C)	C (°C)	D (mm/s)	E (s)	F (%)	S/N_1_ Power Concentration	S/N_2_ Melding Temperature
1	Type I	220	60	65	4.3	20	29.20548	46.85253
2	Type I	220	70	75	4.8	40	25.44311	46.46912
3	Type I	220	80	85	5.3	60	23.81951	46.86169
4	Type I	220	90	95	5.8	80	31.46265	46.8638
5	Type I	220	10	105	6.3	100	30.67761	46.86618
6	Type II	220	70	85	5.8	100	23.101	46.87297
7	Type II	220	80	95	6.3	20	21.63819	46.87698
8	Type II	220	90	105	4.3	40	21.83297	46.88015
9	Type II	220	100	65	4.8	60	20.49977	46.86064
10	Type II	220	60	75	5.3	80	22.70512	46.86709
11	Type III	220	80	105	4.8	80	42.86495	46.87078
12	Type III	220	90	65	5.3	100	37.05373	46.86319
13	Type III	220	100	75	5.3	20	41.05609	46.8677
14	Type III	220	60	85	6.3	40	42.85524	46.86651
15	Type III	220	70	95	4.3	60	42.57005	46.86868
16	Type I	220	90	75	6.3	60	31.62564	46.85823
17	Type I	220	100	85	4.3	80	30.41522	46.86142
18	Type I	220	60	95	4.8	100	29.11894	46.86429
19	Type I	220	70	105	5.3	20	26.63406	46.86636
20	Type I	220	80	65	5.8	40	26.47247	46.85045
21	Type II	220	100	95	5.3	40	21.92641	46.8772
22	Type II	220	60	105	5.8	60	21.5193	46.87977
23	Type II	220	70	65	6.3	80	19.86008	46.86071
24	Type II	220	80	75	4.3	100	22.83108	46.86736
25	Type II	220	90	85	4.8	20	22.4223	46.87324
						MAX	42.86495	46.88015

**Table 11:** Powder concentration and melding temperature results obtained from different optimization methods.

Taguchi Trials		S/N Ratios(dB)			
	Original Design	Taguchi Method (Powder Concentration)	Taguchi Method (Meld Temperature)	GRA	RMCO
**Powder Concentration**	17.080	45.557	21.738	24.067	42.865
**(μ(%), σ)**	(59.952, 0.1313)	(59.998, 0.0049)	(59.700, 0.0300)	(59.967, 0.0531)	(59.999, 0.0071)
**Melding Temperature**	46.049	46.871	46.876	46.864	46.880
**(μ(°C), σ)**	(200.69, 0.3266)	(220.559, 0.5587)	(220.728, 0.2750)	(220.784, 0.1935)	(220.56, 0.5587)

## Data Availability

The original contributions presented in the study are included in the article, further inquiries can be directed to the corresponding author.
